# Peritoneal and Pulmonary Tuberculosis in a Postpartum Female with Elevated Cancer Antigen 125 and Ascites

**DOI:** 10.1155/2022/7012943

**Published:** 2022-10-26

**Authors:** Olga Lavrynenko, Moulika Baireddy, Srilekha Bodepudi, Hector Santos, James Cortez, Olga Zemlianitsyna, Fernando Sanchez

**Affiliations:** ^1^University of the Incarnate Word School of Osteopathic Medicine, Laredo Medical Center, 1700 E Saunders St, Laredo, TX 78041, USA; ^2^Kharkiv, National Medical University, Nauky Av,4, Kharkiv 61000, Ukraine

## Abstract

**Background:**

Peritoneal tuberculosis is a rare form of extrapulmonary tuberculosis and presents a challenging diagnosis because of its nonspecific clinical manifestations. Peritoneal TB mimics other pathologies, including abdominal carcinomatosis, especially when the patient presents with ascites and an elevated cancer antigen (CA)-125 levels. *Case Presentation*. A postpartum 20-year-old Hispanic female recently discharged after transverse cesarean surgery, presented to the ER with fever, chills, edema, abdominal distension, nausea, and vomiting. The patient was febrile, tachycardic, and hypotensive. Chest X-ray demonstrated alveolar and interstitial consolidations; chest CT revealed tree-in-bud opacities in the right lower lobe, suggestive of atypical (TB)/fungal infection. CT of the abdomen and pelvis demonstrated ascites, omental thickening, peritoneal thickening, and mesenteric adenopathy, suggestive of carcinomatosis. She was admitted with a presumed diagnosis of sepsis secondary to pneumonia and started empirically on broad-spectrum antibiotics without clinical improvement. A battery of oncology markers was ordered and revealed a mildly elevated cancer antigen (CA)-125. Diagnostic paracentesis showed lymphocytic predominance with positive mycobacteria PCR, elevated adenosine deaminase (ADA), and no malignant cells. Subsequently, the sputum acid-fast bacilli (AFB) stain returned positive for tuberculosis, confirming the diagnosis of pulmonary tuberculosis. A peritoneal biopsy was obtained and demonstrated caseating granulomas consistent with peritoneal tuberculosis. The patient was started on standard antituberculosis therapy with clinical improvement.

**Conclusions:**

This case highlights the need for a high-level of suspicion for peritoneal tuberculosis in a patient with pulmonary tuberculosis who presents with intra-abdominal ascites, omental thickening, peritoneal thickening, and mesenteric lymphadenopathy, despite the presence of an elevated CA-125 level.

## 1. Introduction

Peritoneal tuberculosis (PT) is a rare form of extrapulmonary tuberculosis (TB). The most common pathway leading to the development of PT is the reactivation of latent TB in the peritoneum via lymphogenous or hematogenous spread of bacteria from the primary pulmonary site of a prior infection. In cases of active pulmonary TB, the peritoneum also can be involved in lymphogenous spread [1]. In rare cases, ingestion of bacilli or unpasteurized milk can cause PT. Another pathway of peritoneal infection is the direct spread of TB from an infected adjacent focus, such as the fallopian tubes [2]. Immunocompromised individuals, including patients with human immunodeficiency virus (HIV), cirrhosis, diabetes mellitus, malignancy treated with immunosuppressive agents, corticosteroids, and patients on continuous peritoneal hemodialysis, are at high risk for a variety of chronic granulomatous diseases, including TB [2, 3]. Tuberculosis involving the peritoneal cavity is an uncommon presentation, however, and should be considered in any individual who presents with abdominal symptoms and who resides in an endemic area where the prevalence of TB is high.

Pregnancy is considered to be an immunosuppressive state; T-helper 1 (Th1) cells and proinflammatory markers are suppressed during pregnancy and can mask the symptoms of TB. In the early postpartum state, women undergo an immune reconstitution period, where the symptoms of TB become manifest as their immune system recover. Early postpartum women are twice as likely to develop TB compared to nonpregnant women [4].

The diagnosis of peritoneal tuberculosis is often delayed because the symptoms at the time of presentation are nonspecific symptoms and the individual is misdiagnosed with peritoneal carcinomatosis or ovarian cancer [5].

## 2. Case

A 20-year-old Hispanic woman, living in Laredo, a city on the Mexican border, presented to the emergency room with complaints of recurrent fever, chills, abdominal distension, bilateral lower extremity swelling, nausea, nonbloody emesis, and watery diarrhea. She was discharged from the hospital one week prior, after undergoing a primary low transverse cesarean section at 37.6 weeks of pregnancy. The delivery was performed without any surgical or medical complications. The patient denied any prior medical conditions, use of alcohol or illicit drugs, exposure to known TB contacts, recent travel to Mexico, and a family history of cancer. There are no sick contacts or animals at home.

On initial presentation, the patient was febrile with a temperature of 102.0°F, had tachycardia with a heart rate of 129 beats per minute, and had a blood pressure of 104/56 mm Hg. Physical examination demonstrated abdominal distension and tenderness, shifting dullness to percussion, a positive fluid wave sign, 3+ bilateral peripheral pitting edema, generalized lymphadenopathy and rales and rhonchi in both lungs. The liver and spleen were not palpable. Jaundice, palmar erythema, and spider angiomatosis were not noted. The laboratory reported a microcytic, hypochromic anemia (Hgb = 9.5 g/dL, Ht = 30.8%) with slight anisopoikilocytosis. The platelet count was 305 × 10^3^/mcL and WBC was 7.92 × 10^3^/mcL with 80% neutrophils and 9.3% lymphocytes. Procalcitonin was elevated at 10.68 ng/ml (normal range 0–0.5 ng/ml). CRP was elevated at 13.39 mg/dl (normal range 0.2–0.9 mg/dl) and ESR was elevated at 29 mm/hr (normal range 0–20 mm/hr). Plasma total protein and albumin were reduced at 4.5 g/dL and 2.33 g/dL, respectively. A 24-hour urine protein collection yielded 0.4 g/24 h. Renal function, liver function, and electrolytes were within normal limits.

**Figure 1:**
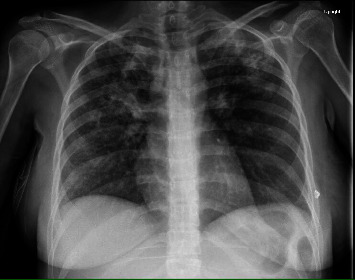
Chest X-ray image showing both alveolar and interstitial infiltrates with findings most prominent within the bilateral upper lobes and right perihilar region.

**Figure 2:**
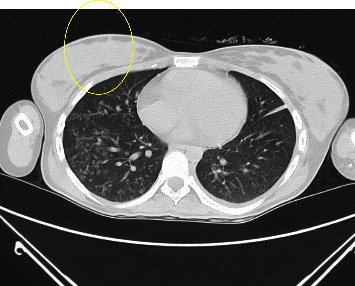
CT chest image demonstrates multiple tree-in-bud opacities within the right lower lobe (yellow circle). Findings can also be seen in the setting of aspiration or other atypical/fungal infections, sarcoidosis, and some viruses such as CMV, among other etiologies.

**Figure 3:**
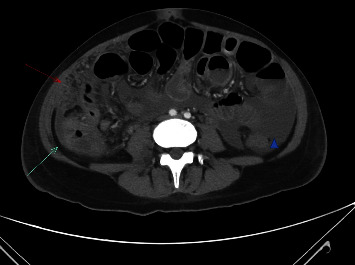
Abdominal findings associated with peritoneal tuberculosis on CT. Omental thickening (red arrow) and peritoneal thickening (green arrow) are noted along with ascites (blue arrowhead).

**Figure 4:**
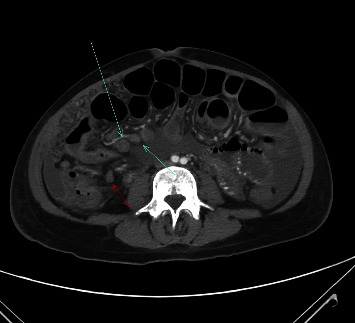
An additional CT image of the abdomen demonstrates mesenteric adenopathy (green and red arrows).

The presenting X-ray demonstrated diffuse alveolar and interstitial opacities in both lungs, with more extensive findings in the upper lobes and right perihilar region, consistent with pneumonia (Figure 1). The CT of the chest showed extensive bilateral infiltrates with a tree-in-bud configuration (Figure 2). A CT scan of the abdomen/pelvis demonstrated ascites, colonic wall thickening, omental thickening, peritoneal thickening, and mesenteric lymphadenopathy, initially read by radiology as suggestive of carcinomatosis and consistent with the elevated CA-125 level (Figures 3 and 4). There was no hepatosplenomegaly or evidence of cirrhosis. A transthoracic echocardiogram demonstrated a left ventricular ejection fraction of 65% with mild mitral and tricuspid regurgitation. There was no pericardial effusion or evidence of left ventricular thrombus.

The patient was admitted with a presumed diagnosis of sepsis secondary to bilateral pneumonia and ascites of undetermined etiology. The patient was started on treatment empirically with broad spectrum intravenous antibiotics, vancomycin, and piperacillin-tazobactam. Because of persistent tachycardia and fever for several days, vancomycin therapy was switched to doxycycline. Blood tests for syphilis, Rickettsia typhi, Rocky Mountain spotted fever, Q fever, CMV, EBV, HIV, and hepatitis were negative. Blood, urine, and vaginal cultures showed no bacterial growth. Autoimmune antibodies and TB QuantiFERON PCR (QIAGEN, LABCORP) were negative.

Because of the presence of mesenteric adenopathy and concern about a malignant process, tests for tumor markers revealed a mildly elevated CA-125 at 199.5 units/mL (normal range 0–35 units/mL) and an elevated alpha fetoprotein (AFP) at 13.67 ng/mL (normal range 0–5.63 ng/mL). Carcinoembryonic Antigen (CEA), CA 15-3, and CA 19-9 were within normal limits at 0.5 ng/mL (normal range 0–4), 27.4 units/mL (normal range <30) and 23.1 units/mL (normal range 0–37 unit/mL), respectively.

The patient underwent a diagnostic paracentesis which showed a lymphocytic predominance. The calculated serum ascitic albumin gradient (SAAG) was greater than 1.1 and the lactate dehydrogenase (LDH) level in the ascitic fluid was elevated at 399 IU/l. Ascitic fluid culture was negative for bacteria, and cytology was negative for malignant cells.

Subsequently, the Mycobacteria PCR of the ascitic fluid was reported to be positive, and the level of adenosine deaminase (ADA) in the ascitic fluid was reported elevated at 34.9 U/l (normal <7.6 U/l). Around this time, the culture for acid-fast bacilli (AFB) in the sputum was reported to be positive.

Because of the high suspicion of peritoneal TB in this patient, antituberculosis therapy (Rifampin, Isoniazid, Pyrazinamide, Ethambutol (RIPE)) was initiated, prior to the return of the AFB cultures. To confirm the diagnosis of peritoneal TB, the patient underwent a diagnostic laparoscopy with a peritoneal biopsy, which demonstrated an inflammatory response with caseating granulomas and a positive AFB stain, confirming the diagnosis of peritoneal tuberculosis.

After 3 weeks of RIPE therapy, the patient's clinical condition improved and CA-125 decreased from 199.5 units/mL to 89.6 units/mL, CRP decreased from 13.39 mg/dL to 2.55 mg/dl and ESR fell from 29 mm/hr to 23 mm/hr.

## 3. Discussion

Peritoneal tuberculosis is a rare manifestation of extrapulmonary TB with an incidence of 6%, according to the CDC [6, 7]. Risk factors for peritoneal TB are immunocompromised status, such as HIV, cirrhosis, diabetes mellitus, malignancy treated with immunosuppressive agents, corticosteroids, continuous peritoneal hemodialysis, and chronic kidney disease [6, 8]. Additionally, as in the present case, pregnancy is also an immunocompromised state. In individuals who present with PT, the female gender is more common than the male gender [8].

Peritoneal TB presents a diagnostic challenge because of its nonspecific clinical presentation, including abdominal pain and distension, fever, weight loss, nausea, and vomiting [1–3]. Consequently, time from the onset of infection to diagnosis can vary from weeks to months or years [6]. Routine hematologic evaluation is nonspecific for the diagnosis of peritoneal TB, and includes normochromic normocytic anemia, thrombocytosis, normal white blood cell count, elevated ESR, and hypoalbuminemia [1, 2, 9]. In our case, the hematology results demonstrated a normal white blood cell count and hypoalbuminemia, but the platelets count was normal a hypochromic, microcytic anemia was present. However, reactive thrombocytosis occurred later during hospitalization.

A positive tuberculin skin test or serum QuantiFERON supports the diagnosis of peritoneal TB, but in the study by Fan et al. The sensitivity for QuantiFERON was only 72% [2, 10]. Further, this test cannot differentiate between latent and active TB [1]. PCR for Mycobacteria TB is highly specific but has low sensitivity [2].

Definitive diagnosis of peritoneal TB involves a diagnostic paracentesis in patients with ascites [2, 3]. A positive AFB smear and culture for Mycobacteria on the ascitic fluid establishes the diagnosis of PT [3]. However, the ascitic fluid AFB smear and cultures are often negative, and because of the slow AFB growth rate, it may take as long as 8 weeks for the culture to become positive [11]. Cell counts from the ascitic fluid analysis usually demonstrate a lymphocytic predominance [3, 9, 12]. The SAAG score on the ascitic fluid in patients with TB without cirrhosis usually is less than 1.1 [2, 12, 13]. However, the SAAG score in our patient was greater than 1.1. This could be explained by the initial diuretic treatment of volume overload and albumin infusion of the early stages of hospitalization.

Elevation of ADA in the ascitic fluid is common in patients with peritoneal TB. It has a sensitivity of 97% and a specificity of 100% [13, 14]. An ADA level > 39 in the ascitic fluid is highly diagnostic of peritoneal tuberculosis [14]. ADA is an aminohydrolase and potent modulator of T-cell differentiation and is found in erythrocytes, lymphocytes, and the cerebral cortex. ADA is increased in effusions resulting from infections, rheumatological diseases, and lymphoproliferative disorders, including TB, and especially peritoneal TB, due to the increase in T-cell differentiation in response to the TB antigen [2, 15].

An elevated LDH level in the ascitic fluid, as found in our patient, is also common in peritoneal TB [2] but is also present in patients with peritoneal carcinomatosis [2].

Ultrasound and CT scans of the abdomen often demonstrate abdominal changes [2, 3], such as ascites (36%–67%), lymphadenopathy (14%–47%), and peritoneal thickening (23%–32%), but these findings are nonspecific [1, 16], and can be observed in patients with abdominal carcinomatosis and other bacterial intra-abdominal infections [1, 3]. However, some radiological findings that, if present, is highly suggestive of peritoneal TB. These include high density ascitic fluid, nodular thickening of the peritoneum and mesentery, splenomegaly, omental infiltration, and lymph node and splenic calcifications [5, 17]. In the study by Sohail et al., [17] the specificity for peritoneal TB with high density ascitic fluid was 66.7%, with omental infiltration it was 78.3%, with splenomegaly it was 33.3%, with splenic calcifications it was 11.6%, and with nodular mesenteric/peritoneal thickening it was 63.8% [5, 17].

Definitive diagnosis of PT requires invasive diagnostic testing [1]. The gold standard is a laparoscopic peritoneal biopsy with pathological and microbiological confirmation [2, 11], especially in patients who do not present with ascites. The sensitivity of peritoneal biopsy is 85–100% [9]. Peritoneal biopsy can reveal a thickened peritoneum with or without yellow-white tubercles, dense adhesions, and caseating granulomas [6]. Characteristic granuloma with central necrosis on biopsy, which was present in our patient, has an estimated sensitivity of 71–100% and a specificity of 100% [6]. In our patient, the admitting diagnosis was bilateral pneumonia with presumed sepsis and ascites of unknown etiology. Because the focus was on pneumonia with sepsis, this resulted in a delay in evaluating the ascites. Because there was no response to broad spectrum antibiotics, workup of the ascites was initiated. The abdominal CT was initially read as consistent with carcinomatosis, the CA-125 test was positive, and the TB QuantiFERON PCR was negative. Subsequent evaluation of the ascitic fluid was negative for malignant cells, but the Mycobacteria PCR test later returned as positive. This was followed by a peritoneal biopsy which definitively established the diagnosis of peritoneal TB.

Antituberculosis treatment should be started before waiting for the biopsy results or ascitic fluid cultures in a patient with positive findings (lymphocytic predominance, positive AFB stain, increased AD and LDH, positive mycobacteria TB on PCR) of peritoneal TB from the diagnostic paracentesis, much like our patient.

Peritoneal TB is occasionally associated with involvement of the ovaries, and this can cause an elevated CA-125 [2, 4–6, 11, 18], which is also sensitive, but nonspecific ovarian tumor marker. CA-125 also can be elevated in pulmonary and extrapulmonary tuberculosis. A CA-125 level greater than 35 u/mL has a sensitivity of 83.3% and a specificity of 50% for PT [2]. This marker has been proposed to follow the response to therapy [5, 18] and to determine if medication resistance is present [19]. In our patient, the CA-125 level decreased from 199.5 units/mL to 89.6 units/mL within 3 weeks of starting RIPE therapy, indicating a good response to therapy.

## 4. Conclusions

Peritoneal tuberculosis is a rare form of extrapulmonary tuberculosis. Because the clinical symptoms are nonspecific, the diagnosis of peritoneal TB is challenging. The diagnosis of peritoneal TB should be suspected in patients with relevant clinical manifestations (fever, weight loss, abdominal pain, distension, ascites, hepatomegaly, and diarrhea) and relevant epidemiologic factors (history of prior TB infection, previous TB exposure, past or present residence in an endemic TB area, travel to an endemic TB area). Radiologic findings in patients with peritoneal TB are nonspecific and include ascites, lymphadenopathy, and peritoneal thickening. In patients with ascites, diagnostic paracentesis should be performed. Analysis of the ascitic fluid usually demonstrates a high lymphocyte count, a positive acid-fast bacilli smear, a positive PCR for Mycobacteria tuberculosis and an elevated ADA and LDH level. An elevated ascitic ADA level is a highly sensitive finding and supports the diagnosis of peritoneal TB. In the absence of ascites, a peritoneal biopsy should be considered.

After establishing the diagnosis or if there continues to be a high clinical suspicion of peritoneal TB, patients should be started on antituberculosis treatment, similar to the treatment of pulmonary TB. In the case of peritoneal TB, if the serum CA-125 is elevated, it can be used to monitor the effectiveness of therapy.

## Data Availability

Data are available from the corresponding author upon reasonable request.
